# Charcoal maker's pneumoconiosis

**DOI:** 10.1002/rcr2.1040

**Published:** 2022-09-14

**Authors:** Hiroki Watanabe, Toshiyuki Sumi, Yoshiko Keira

**Affiliations:** ^1^ Department of Pulmonary Medicine Hakodate Goryoukaku Hospital Hokkaido Japan; ^2^ Department of Respiratory Medicine and Allergology Sapporo Medical University School of Medicine Sapporo Japan; ^3^ Department of Surgical pathology Hakodate Goryoukaku Hospital Hokkaido Japan

**Keywords:** charcoal, occupational hazard, pneumoconiosis

## Abstract

Negligence of health safety is common cause for occupational hazards such as charcoal maker's pneumoconiosis. This case report highlights such a case where the condition developed because of not wearing a dust mask due to the heat during production. As the condition is mostly asymptomatic, preventive measures must be encouraged.

## Graphical abstract

We present a case of charcoal maker's pneumoconiosis that developed due to negligence of health safety measures, that is, not wearing a dust mask, during production. As the condition is mostly asymptomatic, preventive measures must be encouraged.

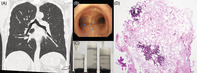

## CLINICAL IMAGE

**FIGURE 1:**
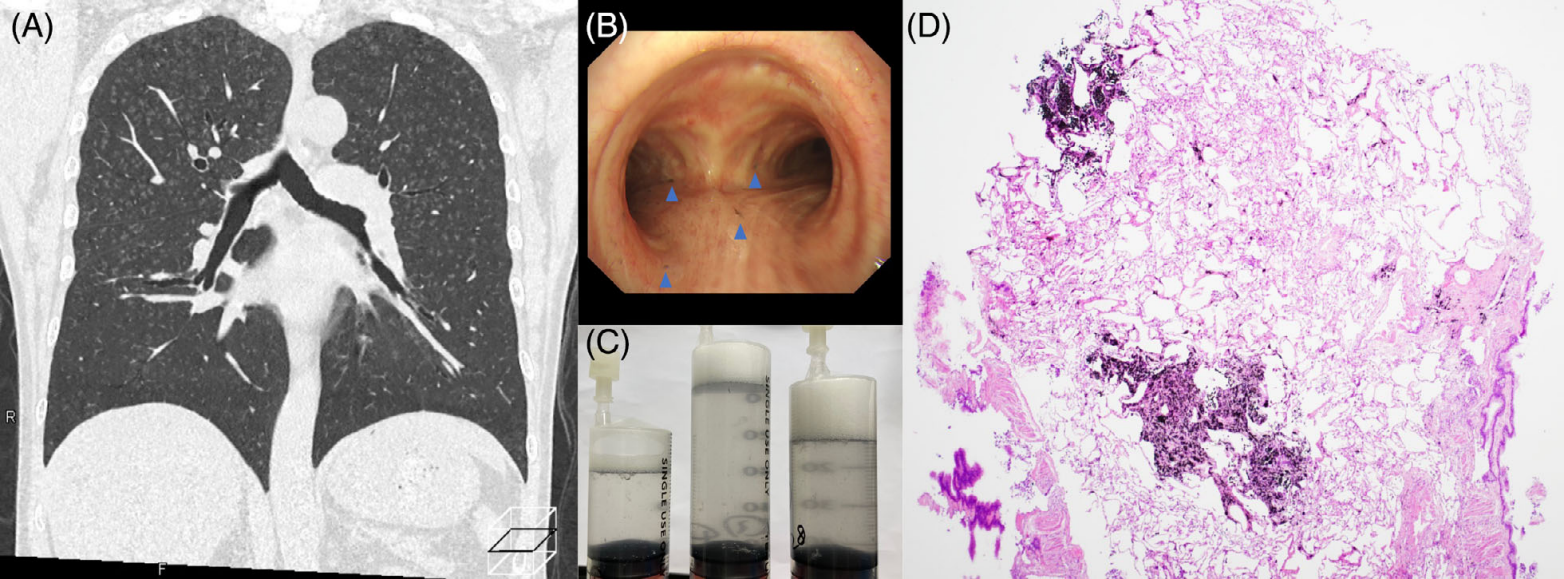
(A) Chest computed tomography showed a lobular central frosted nodule predominantly in the upper lobe. (B) Bronchoscopy revealed coal dust deposits (blue arrowhead indicated) on the bronchial mucosal wall. (C) Bronchoalveolar lavage fluid showed fine black suspended material. (D) Histopathological examination of the lung tissue confirmed lobule‐centric coal dust deposition (haematoxylin and eosin staining, ×2)

A man in his 50s, a charcoal producer, presented with abnormal chest shadows. He had occasional black sputum without dyspnea. Respiratory function tests were within limits. Chest computed tomography showed a lobular central frosted nodule predominantly in the upper lobe (Figure 1A). Although hypersensitivity pneumonitis was suspected, the shadows did not improve with hospitalization for antigen isolation. Bronchoscopy revealed coal dust deposits on the bronchial mucosal wall (Figure 1B), and bronchoalveolar lavage fluid showed fine black suspended material (Figure 1C). Transbronchial cryobiopsy and pathological examination confirmed lobule‐centric coal dust deposition (Figure 1D). He was diagnosed with pneumoconiosis from charcoal burning by not wearing a dust mask during production; he was instructed to ensure wearing a mask. Histopathology revealed no inflammation or fibrosis, and the patient is on follow‐up. There are few reports of pneumoconiosis in charcoal makers.^1^ Charcoal makers have increased dust exposure dependent respiratory symptoms such as cough, sputum, and shortness of breath; however, respiratory function (FVC, FEV1) decreases are less.^2^ Lack of inflammation and fibrosis in the lungs surrounding the charcoal deposition in this case and previous report confirms that this is asymptomatic with little impairment of respiratory function.^1^ Thus, preventive measures should be encouraged for charcoal makers.

## AUTHOR CONTRIBUTION

*Conceptualization*: Toshiyuki Sumi. *Data curation*: Hiroki Watanabe. *Formal analysis*: Yoshiko Keira and Hiroki Watanabe. *Investigation*: Yoshiko Keira. *Roles/writing – original draft*: Toshiyuki Sumi. *Writing – review & editing*: Hiroki Watanabe. All authors have read and approved the final version of the manuscript.

## CONFLICT OF INTEREST

None declared.

## ETHICS STATEMENT

The authors declare that appropriate written informed consent was obtained from the patient for the publication of this report and any accompanying images.

## Data Availability

Data sharing not applicable to this article as no datasets were generated or analysed during the current study.
